# Eleven Letters and Answers

**Published:** 1880-07

**Authors:** 


### Our Correspondence.

HOT SPRINGS, Ark. March 6, 1880.

DEAR DOCTOR :

Think I made you a partial promise of a letter from this famous resort for invalids and tourists from all parts of this country, and from the entire civilized world.

These celebrated Springs are situated in Garland Co., about sixty miles by rail from Little Rock, the capitol of the state. They flow out of what is known as Hot Springs mountains—a spur of the Ozark Range. They flow out of a territory covering some six acres of ground, and have a front on a beautiful dashing creek, of about four hundred yards—into which they used to empty, till diverted by iron pipes, leading to the various bath houses. The rock on the border of the creek, extending back up the mountain from a few yards to one hundred, is made up from the deposit from the springs and is called—tupa.

Just opposite this creek, not more than fifty yards distant, is what is called Cold Spring mountain; out of which flow numerous cold, clear springs of water. They also find their way into the same creek and formerly the hot waters and cold mingled, uninterruptedly. One cannot go in any direction up the little narrow valleys, without running across many springs—either of pure water, or those impregnated with iron or other mineral substances.

South, some six miles, is a spring sending out waters said to contain sulphur and potash; and many, after bathing here in these waters for a time, go there for rest and change—it is said, with benefit. In another direction, about twelve miles distant, is the famous Mountain Valley Spring, the water of which, it is claimed, is decidedly tonic and diuretic. It is now brought here in a tank, by carriage, and retailed to any who desire to purchase.

There are seventy-one well marked hot springs. The temperature of which varies from about ninety to one hundred and fifty-seven degrees. The smaller the flow, the cooler the water. The largest springs send out the hottest water. It is thought that all the chemical waters come from the same source, while the smaller streams cool the most in their transit to the surface from the depths below. The entire amount of hot water discharged each day is estimated at five hundred thousand gallons. It must either take a large amount of fuel, or an intense chemical action, to heat up so much water to so high a point, from the unknown depths from whence they come.

There has been several partial analysis made of the water; but none, as yet, entirely satisfactory and exhaustive. There seems to be only from eight to twelve grains of mineral constituents to the gallon—not more than is usually found in ordinary drinking water.

There are two very fine and large bath houses —built on the land reserved by the government. The proprietors have, of course, to get the privilege of erection of the same. Besides the above mentioned, there are several smaller ones of less pretention. The bathing facilities are ample for the wants of invalids, at the present. There is, of course, no lack for *hot water*. They erect large reservoirs, made of plank, into which hot water is conveyed and left open to the air, by means of which it is cooled, to reduce the temperature sufficiently for baths.

It is the general opinion of the profession here—and these are resident men of ability,—that the waters owe their curative power to *heat* and *electricity*. All agree that the waters are heavily charged with electric force.

So far as my personal experience goes in the use of the baths, I am persuaded that there is a peculiar force present, which is not found in water artificially heated. The glow after the bath continues longer—the blood remains in the hands and feet for hours, giving a pleasant and agreeable feeling. There is a sense of a peculiar warmth—gentle stimulation that is positively luxurious. The after effects strongly resembles the modern electrical bath.

For many years, scarcely any cases came here, except for rheumatic and *specific* troubles. Most of those coming, made a secret visit, so afraid were they of having it known, that letters often came anonymously, or to some other post office in the vicinity.

This fear of being thought to be the victim of specific disease has now passed away.

But now it is fair to say that not more than two-fifths of the cases among men are specific; nor more than one in twenty among women. It seems to be a well authenticated fact that the kind of disease above mentioned, yields more rapidly to medical treatment while using the baths, than by any other process. A *very large amount* of mercurials and iodides are used internally and by inunction, while using the hot baths. It is claimed that medical remedies are diffused through the tissues as antidotes, and then eliminated with great rapidity by the profuse perspiration caused by long use of the baths. From my own observation in treatment of such cases, while using very warm and long continued baths, I am persuaded that the above philosophy is correct. Indeed, one of the principal physicians here said to me, "You can do just as well as I can here—with the use of the facilities you have at your Cure." But be it remembered, that the waters themselves do not by any manner of means, cure *specific diseases.* Patients drink very largely of these hot waters. They are so largely charged with carbonic acid that they do not oppress the stomach, or produce nausea. Diaphonsis is greatly increased, when desired, by imbibing freely of the water while in the bath.

It is useless for the class of cases, of which we have spoken, to come here for a short stay. It takes from three to six months of active medication in conjunction with the baths to make a cure. And after all that can be done, many fail and gradually sink into an untimely grave.

The largest number that come here for treatment are rheumatics; and it is positively asserted that only *chronic* cases are benefited. No one suffering from acute inflammatory rheumatism should come here, since they are made worse by the use of the baths. But if the case be caused by special poison and is chronic, the relief is often very rapid. It is a common thing to see men carried to the baths, who in a few weeks can go themselves. But in most cases long and persistent use of remedies and baths are necessary to effect a cure. Some cases of severe neuralgia yield rapidly—almost magically to the hydropathic treatment alone. Some, but by no means *all,* of the cases of skin disease yield to treatment here.

There is yet, really so little known, of the scientific use of these mineral and thermal waters, that their use is largely empirical; and until we have more accurate knowledge of the real nature, the utmost confidence can not be put in their use, as a therapeutic agent.

It is to be hoped that at no distant day, the government will appoint a scientific corps to make a thorough and complete analysis of the now famous hot springs of Arkansas.

S. O. GLEASON, M. D.

Here follows several complimentary letters about the BISTOURY.

IRVING, N. Y., Dec 14, 1879.

EDITOR BISTOURY :

Please find one dollar inclosed for BISTOURY for present and next year. I find your journal invaluable to me in my practice. It gives me in short crisp articles, all the news contained in the more expensive journals. I like it better than any other medical journal.

S. M. P., M. D.

Thanks Doctor. You will find in the present number condensed articles from almost every important medical journal in the world. We could not use your article on the uses of petrolium, as a medicine, because both sides of the sheet were written upon.

---

EVANSVILLE, IND., Dec. 28, 1879.

THAD S. UP DE GRAFF, M. D.:

Enclosed subscription to the BISTOURY. I take so many journals that I find it inconvenient sometimes to pay for them. But so sharp a knife as you cut with, should be encouraged.

J. W. C., M. D.

---

ORANGE CHEROKEE CO., GA., Feb. 28, 1880.

DR. UP DR GRAFF :

Have you had any experience with the new anæsthetic which I see so favorably noticed in the *Medical and Surgical Reporter*? Has it all the virtues claimed for it? C. S. S., M. D.

The doctor refers to the Hydro-Bromic Ether, an account of the uses of which is given under the proper head.

---

ADRIAN, MICH., Dec. 19, 1879.

DR UP DE GRAFF :

Here's a dollar for that spicy journal of yours. I am pleased with it. Your manner excellent—material choice for both the physician and layman. Long life to you. N. A. B., M, D.

---

SNIPE'S STORE S. C. Feb. 13, 1880.

ED. BISTOURY :

Please inform me where to obtain Ringer's Hand Book of Therapeutics? E. P. S., M. D.

Of Wm. Wood & Co., 27 Great Jones St, New York.

---

From a score more of letters, we select the two following ones, on Camping Out.

BALTIMORE, MD., March 1, 1880.

DEAR BISTOURY:

Upon your recommendation, I purchased a copy of "Bodines" and read it. So have my household. The very devil has been to pay in my family ever since. My two boys, aided and abetted by their mother, are making tents, ordering new fangled double bottomed tin tableware, and constructing other camping fixtures which I recognize as belonging to Bodines. To cap the climax, I find a chunk of salt pork spiked to the window sill, in the kitchen, this morning! The cook, confound her, has read the book too! We propose organizing a camp somewhere on Chesapeake Bay, early in June, and if it pans out half as well as Bodines, you will hear from me again.

Meanwhile, I am a convert to your wiles, and a PROSPECTIVE CAMPER.

"Prospective Camper" is to be congratulated upon his decision to spend his vacation so healthfully and sensibly. Take your Bodines with you, follow its directions, and if you do not all vote as having had a glorious time, write us the reason why.

---

WILLLIAMSPORT, PA, March 12, 1880.

EDITOR BISTOURY:

If I am in time, I just want to tell you that I've read that blamed book of yours—"Bodines." But that isn't the worst of it—so have the children. For the past two days they have been chalking diagrams of tents on the barn floor, and the sewing girl is up to her ears in "duck," unbleached muslin, ropes, marlin, etc., which she is putting together under direction of the youngsters and a copy of Bodines. They declare their intention of "camping out" on the Lycoming, "like Dr. Up de Graff." You keep on fooling with your horseback articles and your camping out books and you will demoralize our town, because I know of at least a half dozen families making tents and preparing to camp out, all in consequence of that book. * * * * *

J. R. S.

Good again. What a jolly party of us there will be on the banks of the glorious Lycoming this June!

ATLANTIC CITY, N. J. March 11, 1880.

DR UP DE GRAFF:

Your plan of publishing numerous formulæ used successfully by eminent practitioners, and quoting the latter as authority, is an excellent feature of the BISTOURY. Send me the journal for a year.

B. R., M. D.

MINERSVILLE, PA., May 20, 1880.

DR. UP DE GRAFF:

We have a little girl eight years old, who has one cross-eye. It turns inward. She has been so ever since she had the measles, three years ago. Can anything be done to remedy the deformity in her eye, and when should it be done? MRS. H.

Take her to an oculist and have her eye straightened. Do not delay the operation longer. She will lose the vision in the crooked eye if you postpone an operation.

---

JOHNSTOWN, N. Y., June 2, 1880.

DR. UP DE GRAFF:

We notice that our son, aged twelve, now attending school, complains of his eyes paining him at times, and that he is inclined to squint, or turn one eye inward occasionally. Is there any danger of his becoming cross-eyed, and can we do anything to prevent such a catastrophy. M.

Your boy is probably over-sighted (hypermetrophic). Send him to an oculist and have his eyes tested and spectacles fitted. In this manner, you may prevent not only cross-eye, but serious impairment of vision, as well.

---

TOPEKA, KANSAS, June 3, 1880.

EDITOR BISTOURY:

The BISTOURY has found its way to my office, and I send you three subscribers from my professional friends. I like your short, crisp medical items and news. I gain a deal of information from them without wading through so much literature to get at the "point." This department of your journal is very skillfully edited, and should bring you hosts of subscribers from among the profession. J. I. D., M. D.

The above speaks for itself and requires no comments, save to thank the Doctor for his good words and practical approval of our journal.

---

The Medical Items and News department of the BISTOURY is a feature intended for the exclusive benefit of the physician in active and busy practice. As the Doctor writes, we read all the important medical journals of the world, and condense into short articles all matters that we think will interest or be of practical benefit to the physician. We call attention of the profession to this very full and valuable department.

